# Surface antigen SAG1 mediates *Toxoplasma gondii* fitness and host cell attachment in IFNγ-stimulated cells

**DOI:** 10.1128/iai.00010-25

**Published:** 2025-07-03

**Authors:** Olivia Cohen, Parag Maru, Qinli Liang, Jeroen P. J. Saeij

**Affiliations:** 1 Department of Pathology, Microbiology and Immunology, School of Veterinary Medicine, University of California Davis 240470, Davis, California, USA; 2 Service de Maladies Infectieuses et Tropicales, CHU Toulouse 36760 , Toulouse, France; Tulane University, New Orleans, Louisiana, USA

**Keywords:** *Toxoplasma*, SAG1, attachment, interferon gamma, surface antigen, sialic acid

## Abstract

*Toxoplasma gondii* is an obligate intracellular protozoan parasite that can establish lifelong infections and cause severe disease in immunocompromised individuals. Interferon gamma (IFNγ) is a key host defense cytokine that induces a variety of toxoplasmacidal mechanisms. Recent CRISPR/Cas9 loss-of-function screens identified multiple *Toxoplasma* genes important for fitness in IFNγ-stimulated cells. One consistent hit in several screens was the parasite surface antigen, SAG1. Here, we used CRISPR/Cas9 to generate a *SAG1* knockout strain and found that SAG1 is important for parasite fitness specifically in IFNγ-stimulated cells. Mechanistic studies revealed that host surface sialic acids are important for parasite attachment, especially in IFNγ-stimulated cells. SAG1-deficient parasites had reduced attachment efficiency, which was exacerbated in IFNγ-treated cells. These findings highlight the role of SAG1 in mediating robust parasite attachment, especially in the context of immune pressure.

## INTRODUCTION

*Toxoplasma gondii* (*T. gondii*), the causative agent of toxoplasmosis, is an obligate intracellular protozoan parasite that belongs to the phylum Apicomplexa (1). It can establish a chronic infection in the human host that is asymptomatic in the vast majority of cases but can cause life-threatening disease in the developing fetus and in immunocompromised individuals (2). The infection is widespread, with a seroprevalence rate of ~25% worldwide (3).

Its success as a pathogen largely depends on its efficient invasion of host cells and its ability to evade the host immune response. Understanding the molecular mechanisms of host cell invasion and immune evasion is important for developing strategies to combat toxoplasmosis. The initial step in *T. gondii* infection involves the recognition and attachment to host cells, a process mediated by the parasite’s surface proteins, including glycosylphosphatidylinositol-anchored surface antigen (SAG) family members and micronemal proteins (MICs) (4, 5). Among the SAG family members, SAG1 (encoded by TGGT1_233460) is one of the most abundant and well-characterized. It is a known immunogen (6) and has been implicated in host cell recognition and attachment (7). Sialic acids (8), particularly alpha2,3-linked sialic acids (9), and sulfated proteoglycans (10), such as heparan sulfate (11) proteoglycans, are thought to serve as receptors for *T. gondii* attachment. Parasite lectins, including several MICs (12), have been shown to interact with hosts' sulfated polysaccharides (13), with this interaction playing a role in parasite invasion. For example, *Tg*MIC1 specifically recognizes sialylated oligosaccharides on host cell surfaces, thereby facilitating attachment and invasion (14). *T. gondii* sialic acid binding protein 1 (SABP1) is another protein that has been shown to bind sialic acid on the surface of host cells (15). Following attachment, the apical microneme and rhoptry organelles secrete the transmembrane adhesin apical membrane antigen 1 (AMA1) and the host cell surface receptor rhoptry neck protein complex (RON complex), respectively, to drive the invasion process to completion (16).

*T. gondii* infection triggers mainly a type 1 immune response, with the main defense mechanism being the secretion of interferon gamma (IFNγ). Upon infection, Toll-like receptors (TLRs) present on innate immune cells are activated and induce the secretion of interleukin (IL)-12. This cytokine promotes T and natural killer (NK) cells to secrete IFNγ, inducing a variety of toxoplasmacidal mechanisms (17). In mice, the effects of IFNγ are predominantly mediated through the induction of immunity-related GTPases (IRGs) and guanylate-binding proteins (GBPs) that destroy the parasitophorous vacuole (PV) and the parasite within (18). In human cells, IFNγ can induce atypical apoptotic cell death in infected human macrophages, but other effectors induced by IFNγ seem to vary depending on cell type and parasite strain (19). Recently, it was shown that the IFNγ-inducible E3 ubiquitin ligase RNF213 (ring finger protein 213) is recruited to the parasitophorous vacuole membrane (PVM) where it mediates ubiquitylation of the PV, recruitment of ubiquitin adaptor proteins, and restriction of *T. gondii* (20, 21). However, most genes involved in *T. gondii*’s evasion of the immune system and survival in response to IFNγ remain uncharacterized.

In recent years, CRISPR/Cas9 screens have facilitated the identification of genes important for *T. gondii*’s survival under immune pressure. Since IFNγ secretion is a major immune defense pathway used by the human host against *T. gondii*, we and others recently performed CRISPR/Cas9 screens to identify genes conferring *T. gondii* fitness in IFNγ-stimulated human foreskin fibroblasts (HFFs) (22, 23). These screens yielded several promising hits, including SAG1. Additional screens in IFNγ-stimulated murine bone marrow-derived macrophages (BMDM) (24), as well as several *in vivo* screens, also identified SAG1 as important for parasite survival under immune stimulation (25, 26). Here, we investigated the role of SAG1 in conferring parasite fitness in IFNγ-stimulated cells. By generating a *T. gondii SAG1* knockout strain and assessing parasite fitness in naive and IFNγ-stimulated cells, we confirm that SAG1 is important for parasite survival under immune pressure. In IFNγ-stimulated cells, the absence of SAG1 led to significantly decreased attachment, suggesting IFNγ modulates the host cell in a way that affects the parasite attachment mechanism.

## RESULTS

### SAG1 knockout parasites are more susceptible to IFNγ compared to wild-type parasites

**Fig 1:**
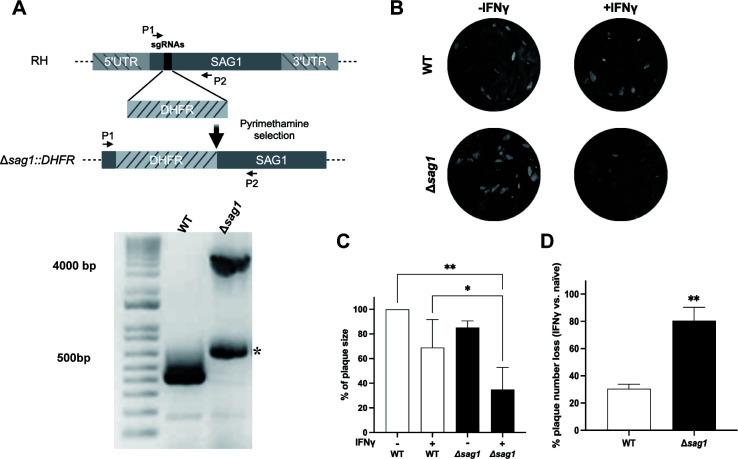
SAG1 knockout parasites are more susceptible to IFNγ. (**A**) Schematic illustration showing the CRISPR strategy to disrupt the *SAG1* gene with an *DHFR* selection cassette. Agarose gel electrophoresis of PCR-amplified products using primers P1 and P2 specific for SAG1. In the Δ*sag1* strain, the *DHFR* cassette insertion results in a ~4 kb product, while the wild-type strain lacks this insertion, producing the expected 467 bp product. * Aspecific band. (**B and C**) The percent reduction in the mean plaque area (representative images in B) relative to the plaque area of wild-type parasites in naive HFFs is shown for wild-type and Δ*sag1* strains in both naive and IFNγ-stimulated (10 units/mL) HFFs. Data are mean ± SD from three independent experiments, and statistical differences were analyzed by a two-way ANOVA (*P < 0.05, **P < 0.01). Data represent the means ± SD from three independent experiments. (**D**) The percentage decrease in the number of lysis plaques formed in IFNγ-stimulated (10 units/mL) HFFs relative to naive HFFs is shown for each strain. Data represent the means ± SD from three independent experiments. Statistical analysis was determined using one-way ANOVA (**P < 0.01).

Our previous genome-wide loss-of-function CRISPR screen identified 11 high-confidence *T. gondii* genes as contributing to parasite fitness in IFNγ-stimulated HFFs (22). Among these was TGGT1_233460 (SAG1). To confirm this finding, a *SAG1* knockout strain was generated in the *T. gondii* RH strain background using CRISPR/Cas9. After pyrimethamine selection, clonal lines of the knockout parasites were screened and confirmed with PCR (Fig. 1A).

Once the SAG1 knockout strain was confirmed, plaque assays were done to compare the overall growth of wild-type and Δ*sag1* parasites in naive and IFNγ-stimulated HFFs. In naive HFFs, the plaque areas were not significantly different between wild-type and Δ*sag1* parasites. In IFNγ-stimulated HFFs, the plaque areas formed by the Δ*sag1* strain were significantly smaller than those formed by the wild-type (WT) strain. Specifically, in IFNγ-stimulated HFFs, the plaques formed by the Δ*sag1* strain were only 34.8 ± 18% the size of plaques formed by the WT parasites in naive HFFs. In contrast, the WT strain formed plaques that were 68.9 ± 22.8% the size of those in naive HFFs under the same IFNγ-stimulated conditions (Fig. 1B). The Δ*sag1* strain also showed a significantly greater reduction in plaque numbers (80.5 ± 9.7%) compared to the WT strain (30.5 ± 3.3%) when comparing the number of plaques formed in IFNγ-stimulated vs. naive HFFs (Fig. 1C). Thus, the Δ*sag1* parasites have a fitness defect specifically in IFNγ-stimulated HFFs.

### The phenotype observed for Δ*sag1* is independent of cholesterol

**Fig 2:**
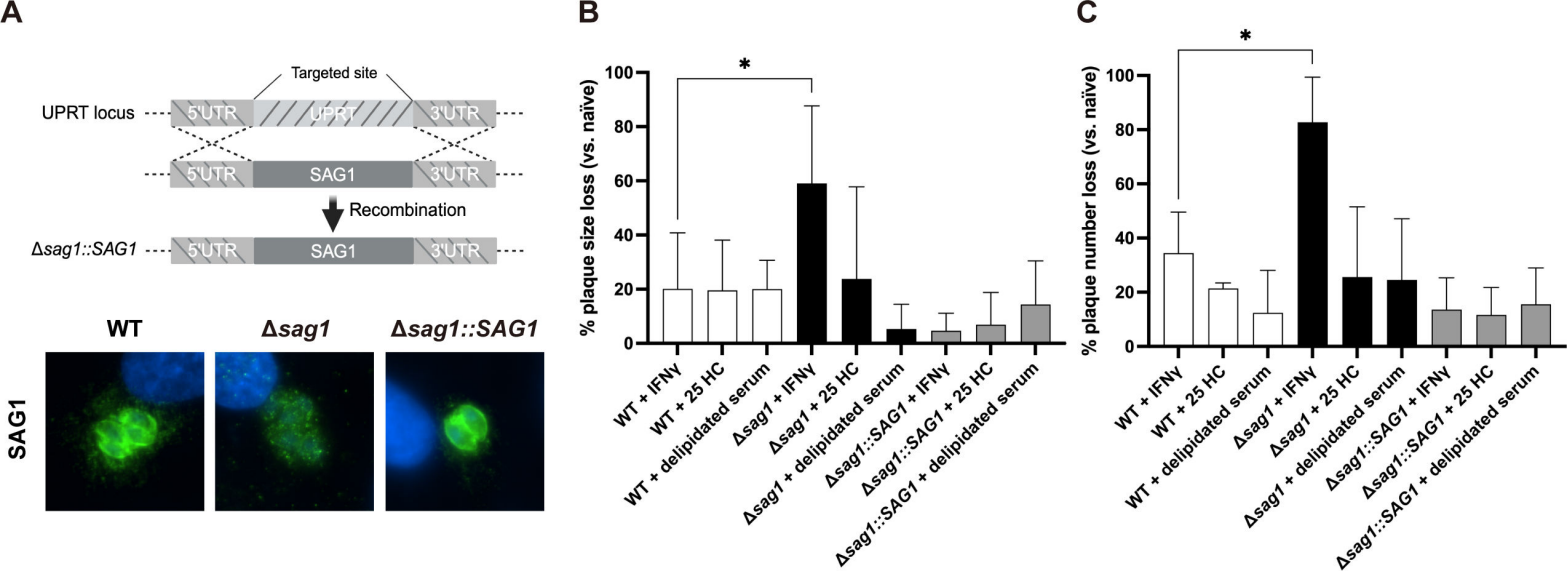
Effect of 25HC, IFNγ, and delipidated serum on the lysis plaque formation in wild-type, Δ*sag1*, and complemented *Toxoplasma* strains. (**A**) Complementation strategy of SAG1 into the *UPRT* locus. An antibody against SAG1 (green) was used to confirm complementation. DAPI stain (blue) shows the nuclear DNA. (**B**) The mean reduction in the lysis plaque area was assessed for infections of naive HFFs compared to HFFs pre-stimulated with 2 µM 25HC or 10 units/mL IFNγ. Additionally, the mean percentage decrease in the lysis plaque area was compared between infections in regular and delipidated sera. Data are shown for three *T. gondii* strains (WT, Δ*sag1*, and Δ*sag1::SAG1*). Statistical significance was determined using two-way ANOVA (**P < 0.01). (**C**) The percentage reduction in the number of lysis plaques formed in HFFs stimulated with IFNγ, treated with 25HC, or cultured in delipidated serum was compared to naive HFFs in regular serum for the indicated strains. Results represent three biological replicates. Statistical significance was determined using two-way ANOVA (**P < 0.01).

To confirm the phenotype observed for the Δ*sag1* strain under IFNγ stimulation, a complemented strain was generated. The complementation was achieved by targeting the *UPRT* locus using two plasmids: one containing the *SAG1* gene driven by its native promoter, and the other expressing Cas9 along with an sgRNA targeting *UPRT*. After transfection, selection with 10 µM FUDR was performed, and successful complementation was confirmed with IFA (Fig. 2A).

Plaque assays were performed to further characterize the phenotype in IFNγ-stimulated HFFs as well as additional conditions. Since IFNγ is known to upregulate cholesterol-25-hydroxylase (*CH25H*) (27), which synthesizes 25-hydroxycholesterol (25HC), we hypothesized that the fitness loss observed in the Δ*sag1* strain could be due to cholesterol depletion. 25HC inhibits cholesterol synthesis and reduces accessible cholesterol at the plasma membrane (28), a mechanism that has been shown to inhibit the entry of several intracellular pathogens (29). For *T. gondii*, host plasma membrane cholesterol plays an important role in parasite invasion (30). The depletion of host membrane cholesterol has been shown to reduce the formation of evacuoles, the vesicles containing rhoptry proteins secreted during host cell attachment, suggesting that cholesterol is important for rhoptry secretion (31).

To test whether the Δ*sag1* phenotype was due to the IFNγ-mediated formation of 25HC and subsequent reduced host cell membrane cholesterol, plaque assays were performed under four conditions: (i) unstimulated HFFs; (ii) IFNγ-stimulated HFFs; (iii) HFFs treated with 25HC; and (iv) HFFs cultured in medium containing delipidated serum (Fig. 2B and C).

In IFNγ-stimulated HFFs, the Δ*sag1* strain showed the previously observed phenotype with a significant reduction in both plaque number and area compared to the unstimulated condition. However, 25HC treatment or the use of delipidated serum did not significantly affect the plaque formation by the Δ*sag1* strain, disproving the initial hypothesis. As expected, the complementation of the Δ*sag1* strain with *SAG1* restored the wild-type phenotype, confirming that SAG1 is important for IFNγ resistance, but that the observed phenotype is independent of cholesterol availability.

### The Δ*sag1* strain has an attachment defect that is exacerbated in IFNγ-stimulated host cells

**Fig 3:**
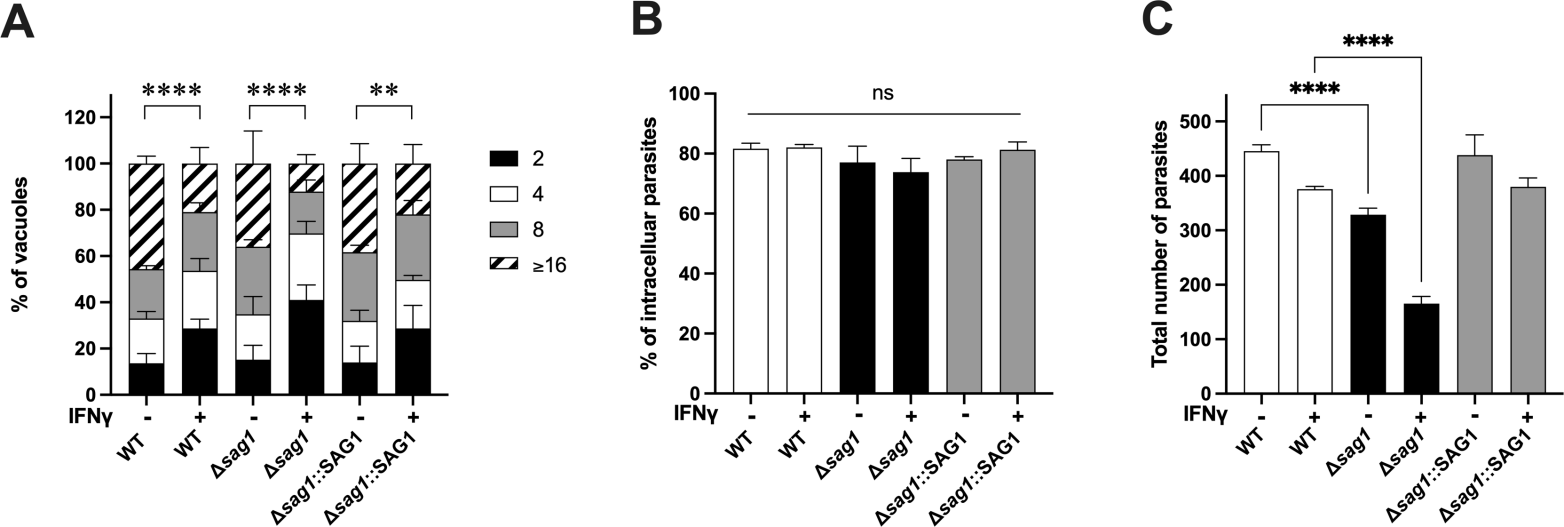
Investigating the mechanisms underlying the Δ*sag1* phenotype. (**A**) HFFs were either prestimulated with IFNγ (10 units/mL) or left unstimulated, followed by infection with the indicated parasite strains (RH background) for 28 h. Cells were fixed, and the number of parasites per vacuole was counted. Statistical analysis using a two-way ANOVA revealed no significant differences between the strains. (**B**) The percentage of intracellular parasites was determined for the three strains (WT, Δ*sag1*, and Δ*sag1::SAG1*) after a 1 h infection at a multiplicity of infection (MOI) of 1 on HFFs pre-stimulated (10 units/mL) or not with IFNγ. (**C**) The total number of parasites was quantified by counting 25 fields of view per strain. The same number of parasites was added to each well for each strain. All the results represent three biological replicates. Statistical significance was determined using a two-way ANOVA (**P < 0.01, ****P < 0.0001).

Since the effect of IFNγ on the growth of the Δ*sag1* strain did not appear to be mediated through IFNγ-mediated upregulation of 25HC, alternative mechanisms were investigated. A parasite per vacuole assay was performed to assess parasite replication during a 28-h infection. The average number of parasites per vacuole was quantified 28 h post-infection in naive and IFNγ-stimulated HFFs (Fig. 3A). Although IFNγ inhibited parasite replication in all three strains, no significant differences were observed among the three strains (WT, Δ*sag1*, and Δ*sag1::SAG1*), suggesting that parasite replication is not affected by SAG1 deletion. Next, the possibility of a defect in parasite entry into host cells was evaluated using an invasion assay. Following a 1-h infection in naive or IFNγ-stimulated HFFs, the percentage of parasites that were extracellular vs. intracellular was determined. In naive HFFs, there was no significant difference in the percentage of intracellular parasites between all the strains, while in IFNγ-stimulated HFFs, the Δ*sag1* strain showed an 8% reduction in the relative number of intracellular parasites compared to WT (Fig. 3B). When the total number of parasites (extracellular and intracellular) was counted, a 26% reduction in total parasites was observed for the Δ*sag1* strain in naive HFFs (Fig. 3C). This reduction increased to 56% in IFNγ-stimulated HFFs. The complementation of the Δ*sag1* strain with SAG1 (Δ*sag1::SAG1*) restored the total number of parasites to wild-type levels. These findings suggest that the phenotype of the Δs*ag1* strain is associated with an attachment defect, which is significantly exacerbated in the presence of IFNγ.

### Sialic acids are important for parasite attachment, especially in IFNγ-stimulated cells

**Fig 4:**
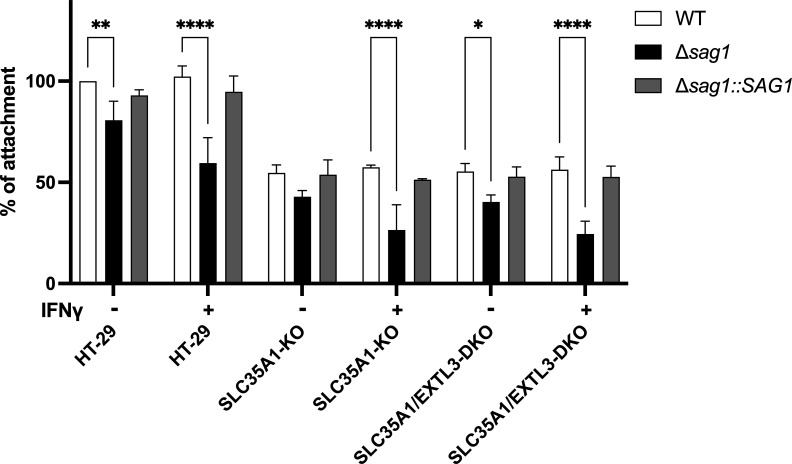
Attachment of *Toxoplasma* is associated with sialic acids. Attachment assay results for the indicated strains (WT, Δ*sag1*, Δ*sag1::SAG1*) were evaluated in HT-29 wild-type cells or in *SLC35A1* single knock-out (SLC35A1-KO) or SLC35A1/exostosin-like glycosyltransferase three double knock-out (SLC35A1/EXTL3-DKO) cells with or without IFNγ stimulation (10 units/mL). Data are presented as mean ± SD from three independent experiments, with 15 fields of view counted per replicate. Statistical analysis was performed using a two-way ANOVA (*P < 0.05; **P < 0.01; ****P < 0.0001).

The previous results suggest that SAG1 is important for parasite fitness by mediating *T. gondii* attachment, particularly under IFNγ stimulation. To test this hypothesis, we performed attachment assays to evaluate the role of SAG1 and host glycan modifications in *T. gondii* adhesion to host cells under both baseline and IFNγ-stimulated conditions. Compared to WT parasites in the unstimulated human HT-29 epithelial cell line (set at 100% attachment), the Δ*sag1* strain displayed a significantly reduced attachment, with only 81% of WT attachment levels, indicating the important role of SAG1 in mediating adhesion. The complementation of the Δ*sag1* strain with SAG1 (Δ*sag1::SAG1*) restored attachment to near-WT levels, confirming the importance of SAG1. In IFNγ-stimulated HT-29 cells, the attachment of WT and Δ*sag1::SAG1* parasites was not significantly different compared to unstimulated cells, whereas that of the Δ*sag1* strain was significantly reduced to 60% of WT levels, indicating that SAG1-independent adhesion mechanisms are less effective under IFNγ stimulation. To further examine the role of specific host glycans, we performed attachment assays using wild-type HT-29, SLC35A1-KO, and SLC35A1/EXTL3-DKO HT-29 cells (32). Solute Carrier Family 35 Member A1 (SLC35A1) encodes a cytidine monophosphate (CMP)-sialic acid transporter essential for sialic acid biosynthesis, while exostosin-like glycosyltransferase 3 (EXTL3) is required for the initiation of heparan sulfate chain synthesis (33, 34). In the absence of IFNγ, the attachment of WT parasites to SLC35A1-KO cells was significantly reduced to 55% of attachment seen in HT-29 cells, indicating dependency on sialic acid. Similar attachment levels were observed in SLC35A1/EXTL3-DKO cells (55% of attachment seen in HT-29 cells), with no further decrease compared to SLC35A1-KO, suggesting that heparan sulfate plays a minimal role in SAG1-mediated adhesion. The Δ*sag1* parasites had even lower attachment rates in SLC35A1-KO, attaching at only 43% of the attachment seen of WT parasites to HT-29 cells, with no further decrease observed in SLC35A1/EXTL3-DKO cells. This further demonstrates that SAG1 and sialic acid are critical for adhesion. Under IFNγ stimulation, the attachment of the WT and Δ*sag1::SAG1* parasites in both SLC35A1-KO and SLC35A1/EXTL3-DKO cells remained stable at approximately 57% of the attachment seen in HT-29 cells, but that of the Δ*sag1* parasites was significantly reduced to ~25% of attachment seen for the WT parasites in HT-29 cells (Fig. 4). Overall, these results highlight the dominant role of SAG1 in ensuring robust parasite attachment, especially when host glycan availability is limited or when host cells are stimulated with IFNγ.

## DISCUSSION

IFNγ is a key mediator of host immune responses against *T. gondii*, activating multiple toxoplasmacidal pathways that restrict parasite survival. Genome-wide CRISPR screens identified the major surface antigen SAG1 as important for parasite fitness under IFNγ stimulation. Using a SAG1 knockout strain (Δ*sag1*) and performing mechanistic studies, we demonstrate that SAG1 plays a major role in mediating robust parasite attachment to IFNγ-stimulated host cells.

IFNγ restricts *T. gondii* at several stages. In murine cells, post-invasion defenses include immunity-related GTPases (IRGs) and guanylate-binding proteins (GBPs) that assemble on the PV and destroy the parasite, whereas human cells rely on RNF213-mediated ubiquitylation and IDO-dependent tryptophan depletion. Our data place SAG1 at an earlier checkpoint: attachment to the IFNγ-remodeled host plasma membrane. IFNγ has been shown to be able to alter this surface by downregulating or redistributing entry receptors, including sialic acid clusters, CD4, and claudin-1, and by modifying the expression and structure of host glycans (35–37). These changes broadly restrict the entry of intracellular pathogens and likely impair low-affinity, SAG1-independent adhesion pathways.

Although IFNγ can increase the sulfation of heparan sulfate proteoglycans via induction of N-deacetylase/N-sulfotransferase (NDST), attachment assays using SLC35A1/EXTL3 double-knockout host cells showed only a minor reduction in binding compared to the SLC35A1 single knockout. Instead, IFNγ-driven host cell plasma membrane remodeling appears to primarily disrupt SAG1-independent adhesion mechanisms, which rely on weaker interactions, making SAG1 indispensable for high-affinity attachment. Accordingly, the Δ*sag1* parasites show a pronounced defect in host cell attachment under IFNγ treatment, whereas the wild-type parasites do not. Loss of surface sialic acids in SLC35A1-deficient HT-29 cells further reduces attachment, especially in the absence of SAG1. These observations indicate that SAG1 functions as a high-affinity adaptor that maintains efficient invasion when alternative adhesins are suppressed by IFNγ. By enabling parasite entry despite cytokine pressure, SAG1 complements post-invasion effectors, such as ROP18 and ROP5. Together, the data support a model in which SAG1 safeguards the first step of invasion under immune stress by stabilizing parasite-host interactions at the remodeled host cell surface.

These findings highlight SAG1 as a potential therapeutic target. SAG1 is highly conserved among the major clonal lineages of *T. gondii*, and orthologs, such as NcSAG1, in *Neospora caninum* play similar roles in host-cell binding and virulence (38). Vaccines directed against SAG1 could, therefore, be used as broad adjunct therapies to current treatments for severe or congenital toxoplasmosis and help prevent *Neospora* infections in cattle. Neutralizing monoclonal antibodies targeting conformational SAG1 epitopes inhibit tachyzoite attachment *in vitro* and may be even more effective *in vivo*, where IFNγ suppresses alternative adhesins and increases parasite reliance on SAG1. Recombinant and DNA-encoded SAG1 vaccines have already reduced brain cyst burden and vertical transmission by up to 60% in murine models. Further work dissecting SAG1-host receptor interactions in IFNγ-stimulated cells may yield additional intervention strategies.

## MATERIALS AND METHODS

### Cell and parasite culture

HFFs were grown in Dulbecco’s Modified Eagle Medium (DMEM) containing 10% fetal bovine serum (FBS), 100 U/mL penicillin/streptomycin, 2 mM L-glutamine, and 10 µg/mL gentamicin (HFF medium). For plaque assays, HFFs were seeded into 24-well plates and infected after cells were confluent. HFFs used for indirect immunofluorescence assays (IFA) were seeded on 12 mm glass coverslips in 24-well plates and infected after cells were confluent. The HT-29, SLC35A1-KO, and SLC35A1/EXTL3-DKO cells were a gift from Dr. Siyuan Ding. The HT-29 cells were maintained in advanced DMEM/F12 supplemented with 1 mM sodium pyruvate, 1× nonessential amino acids, 10 mM HEPES, 100 U/mL penicillin/streptomycin, and 10% FBS and incubated at 37°C with 5% CO_2_. The generation of the SLC35A1-KO and SLC35A1/EXTL3-DKO cells was previously described (32). Parasites were maintained on confluent HFF monolayers in DMEM with 1% FBS, 100 U/mL penicillin/streptomycin, and 2 mM L-glutamine.

### Generation of Δ*sag1* parasites

Knockouts of *TGGT1_233460* (*SAG1*) were generated using the CRISPR/Cas9 system. Two sgRNAs were designed (sequences listed in Table S1). Each sgRNA was inserted into the *BsaI* site of the pU6-Universal vector (which also contains Cas9) (39) using T4 DNA ligase. The parental strain RH-Luc/Δ*hxgprt* (40) was used to generate the knockout. The parasites were co-transfected with the pU6-Universal vector containing both the sgRNA and Cas9 to generate Cas9-directed double-stranded DNA breaks and a template containing a *DHFR* cassette (22) to repair the breaks. The transfected parasites were selected 24 h after transfection with 0.1 µL/mL pyrimethamine to enrich for those carrying the *DHFR* cassette at the Cas9 cut site. After three rounds of drug selection, single clones were isolated using limiting dilution. Once clonal lines were established, they were screened to confirm the insertion of the DHFR repair cassette using primers P1 and P2 listed in Table S1. For the confirmation of knockout, we used genomic PCR, as well as IFA, using a rabbit anti-SAG1 antibody (polyclonal rabbit anti-SAG1 antibodies were a kind gift from John Boothroyd, Stanford University School of Medicine, Stanford, CA).

### Complementation of the Δ*sag1* strain with SAG1

The Δ*sag1* strain was complemented by inserting the *SAG1* gene into the *UPRT* locus. To construct the complementation plasmid, the pUPRT::DHFR-D plasmid backbone (Addgene: plasmid #58528) containing *UPRT* 5′ and 3′ homology regions was PCR-amplified with primers P4 and P5 to remove the *DHFR* cassette. The *SAG1* coding region, including 5′UTR (1,000 bp upstream of the start codon, containing the putative promoter) and 3′UTR (500 bp downstream of the stop codon), was amplified with primers P6 and P7 (Table S1). The amplified plasmid backbone and the amplified *SAG1* insert were assembled using the Gibson assembly reaction and subsequently transfected into the RH-Luc/Δ*hxgprt*/Δ*sag1*/*DHFR+* strain. To facilitate the integration of the *SAG1* expression cassette at the *UPRT* locus through homologous recombination, the plasmid pSAG1::Cas9-U6::sgUPRT (Addgene plasmid #54467) containing an sgRNA targeting *UPRT* was co-transfected. After transfection, the parasites were allowed to replicate and lyse out. Selection with 10 µM FUDR (41) was performed, and after three rounds of selection, limiting dilution was used to isolate single clones. These clones were then screened for SAG1 expression using IFA.

### Immunofluorescence assay (IFA)

HFFs grown on glass coverslips were infected with parasites for 18 to 24 h. After infection, the coverslips were washed with PBS and fixed with 4% formaldehyde for 20 min. Following fixation, the coverslips were washed again with PBS, permeabilized, and blocked using a blocking buffer containing PBS with 3% (w/v) bovine serum albumin (BSA), 5% (v/v) goat serum, and 0.1% Triton X-100. Samples were incubated with the primary antibody at 4°C for 3 to 12 h depending on the specific antibody used. After primary antibody incubation, the coverslips were washed three times with PBS, and fluorescently labeled secondary antibodies along with 4′,6-diamidino-2-phenylindole (DAPI) (for DNA staining) were added. The samples were then incubated for 1 h at room temperature. The coverslips were mounted with a VectaShield mounting medium and visualized using a Nikon Eclipse Ti-S inverted fluorescence microscope equipped with NIS-Elements software (Nikon) and a digital camera (CoolSNAP EZ; Roper Scientific). Imaging was performed using fluorescence, phase contrast, or differential interference contrast (DIC) imaging.

### Plaque assay

Confluent monolayers of HFFs were cultured in 24-well plates. DMEM medium with 10% FBS was replaced 24 h prior to infection. Depending on the experimental condition, the medium was replaced with a medium containing human IFNγ (10 U/mL), 25-hydroxycholesterol (25HC, 2 µM), or 10% delipidated serum. Each well was then infected with 100 parasites using either naive HFFs or HFFs pre-stimulated with IFNγ, 25HC, or 10% delipidated serum. On day five post-infection, plaques were counted and imaged using a Nikon TE2000 inverted microscope equipped with a Hamamatsu ORCA-ER digital camera and a 4× objective. All experiments were performed at least three times with duplicate wells for each condition.

### Parasites/vacuole assay

HFFs containing parasites were harvested and lysed using syringes, and extracellular parasites were washed and counted. A multiplicity of infection (MOI) of 0.1 for each parasite strain (WT, Δ*sag1*, and Δ*sag1::SAG1*) was used to infect coverslips in 24-well plates containing a monolayer of HFFs. The HFF monolayers were either naive or pre-stimulated with 10 U/mL IFNγ for 24 h before infection. Following infection, the plates were incubated at 37°C in a CO_2_ incubator for 28 h. After incubation, the coverslips were washed with PBS, fixed, and processed for IFA using SAG2A antibodies. A total of 200 vacuoles per coverslip were used to count parasites per vacuole, and data were plotted using GraphPad Prism.

### Invasion assay

Parasites were harvested, filtered, and used at an MOI of 1 for each parasite strain (WT, Δ*sag1*, and Δ*sag1::SAG1*). The parasites were added to coverslips in 24-well plates containing monolayers of HFFs, which were either naive or stimulated with 10 U/mL IFNγ for 24 h before infection. The plates were centrifuged at 900 ×*g* for 3 min to synchronize parasite attachment and subsequently incubated at 37°C in a CO_2_ incubator. At 1 h post-infection, the medium was aspirated, and the coverslips were fixed with 4% formaldehyde. The fixed coverslips were washed and incubated with a non-permeabilizing blocking buffer (2% FBS in PBS) for 1 h. To stain extracellular parasites, the coverslips were incubated with the first primary antibody against SAG2A (rabbit 1:2,000) for 3 h at 4°C. After five PBS washes, the coverslips were blocked with a permeabilizing blocking buffer (PBS with 3% [w/v] BSA, 5% [v/v] goat serum, and 0.1% Triton X-100). All parasites (extracellular and intracellular) were then stained using anti-*Toxoplasma* serum obtained from pre-immunized mice for 3 h. The coverslips were washed five times, followed by incubation with secondary antibodies (anti-rabbit Alexa-488 for extracellular parasites and anti-mouse Alexa-594 for all parasites) for 1 h at room temperature. The coverslips were mounted, and 25 fields per coverslip were analyzed. The percentage of intracellular parasites (not green) was calculated for the invasion assay. The total number of parasites for each strain was compared to assess general attachment/invasion efficiency, as unattached parasites would have been removed during washing steps.

### Attachment assay

The attachment ability of tachyzoites from WT, Δ*sag1*, and Δ*sag1::SAG1* strains to various host cell lines was determined by the attachment assays. Freshly egressed tachyzoites (1 × 10^6^) were inoculated onto confluent monolayers of the human colorectal adenocarcinoma cell line HT-29, SLC35A1 single knockout HT-29 cells (SLC35A1-KO), or SLC35A1/EXTL3 double knockout HT-29 cells (SLC35A1/EXTL3-DKO). The host cells were either untreated or pre-stimulated with 10 U/mL IFNγ for 24 h before infection. Following the addition of parasites, the plates were incubated at 37°C and 5% CO_2_ for 15 min. Non-attached parasites were removed by washing the cells with PBS. To visualize the attached tachyzoites, IFA was performed. The cells were fixed and stained with a primary antibody against SAG2A (rabbit, 1:2,000), followed by an Alexa Fluor 594-conjugated secondary antibody (goat anti-rabbit, 1:2,000). The parasites in 15 random microscopic fields per sample were counted, and each experiment was performed in triplicate.

### Statistical analysis

All statistical analyses were performed using GraphPad Prism. For comparisons involving two variables (e.g., parasite strain and treatment condition), two-way analysis of variance (ANOVA), followed by Tukey’s multiple-comparison test was used. One-way ANOVA was applied when comparing multiple groups under a single condition. For all tests, *P*-values < 0.05 were considered statistically significant. Data are presented as mean ± standard deviation (SD) from at least three independent biological replicates unless otherwise indicated.

## SUPPLEMENTAL MATERIAL

Table S1 iai.00010-25-s0001.docx List of primers used.

ASM does not own the copyrights to Supplemental Material that may be linked to, or accessed through, an article. The authors have granted ASM a non-exclusive, world-wide license to publish the Supplemental Material files. Please contact the corresponding author directly for reuse.
