# Supplementary material for: Surface antigen SAG1 mediates Toxoplasma gondii fitness and host cell attachment in IFNγ-stimulated cells
Source: Infect Immun. 2025 Jul 3;93(8):e00010-25. doi: 10.1128/iai.00010-25 (PMC12341370; doi:10.1128/iai.00010-25)
Supplement: Table S1 — List of primers used. [file iai.00010-25-s0001.docx]

| Primer | Sequence | Use |
| --- | --- | --- |
| P1 | ATGTCGGTTTCGCTGCACCA | Fw primer to confirm the SAG1 knockout |
| P2 | GTCTGCGTTGTCACGGGGAAC | Rv primer to confirm the SAG1 knockout |
| G1 | aagttGAGACGCGCCGTCACGGCAGg | Fw primer to construct the gRNA n°1 and insert it into the specific CRISPR plasmid for SAG1 knockout |
| G2 | aaaacCTGCCGTGACGGCGCGTCTCa | Rv primer to construct the gRNA n°1 and insert it into the specific CRISPR plasmid for SAG1 knockout |
| P4 | GACAGACCGCTGACGGAATC | Fw primer to amplify the pUPRT::DHFR-D plasmid backbone for complementation |
| P5 | AGAAGCCCTGTGGACAGGTC | Rv primer to amplify the pUPRT::DHFR-D plasmid backbone for complementation |
| P6 | gacctgtccacagggcttctTAAACGATCCGGGACGACAC | Fw primer to amplify SAG1 with UTRs and insert it into plasmid for complementation |
| P7 | gattccgtcagcggtctgtcTGACAGGTTCGTCGGGGC | Rv primer to amplify SAG1 with UTRs and insert it into plasmid for complementation |

**Supplementary Table 1 - List of primers used**
